# Ultra-deep sequencing reveals pre-mRNA splicing as a sequence driven high-fidelity process

**DOI:** 10.1371/journal.pone.0223132

**Published:** 2019-10-03

**Authors:** Derrick J. Reynolds, Klemens J. Hertel

**Affiliations:** Department of Microbiology and Molecular Genetics, University of California, Irvine, California, United States of America; Florida Atlantic University, UNITED STATES

## Abstract

Alternative splicing diversifies mRNA transcripts in human cells. While the spliceosome pairs exons with a high degree of accuracy, the rates of rare aberrant and non-canonical pre-mRNA splicing have not been evaluated at the nucleotide level to determine the quantity and identity of these events across splice junctions. Using ultra-deep sequencing the frequency of aberrant and non-canonical splicing events for three splice junctions flanking exon 7 of SMN1 were determined at single nucleotide resolution. After correction for background noise introduced by PCR amplification and sequencing steps, pre-mRNA splicing was shown to maintain a low overall rate of aberrant and non-canonically spliced events. Several previously unannotated splicing events across 3 exon|intron junctions in SMN1 were identified. Mutations within SMN exon 7 were shown to affect splicing fidelity by modulating RNA secondary structures, by altering the binding site of regulatory proteins and by changing the 5’ splice site strength. Mutations also create a truncated SMN1 exon 7 through the introduction of a *de novo* non-canonical 5’ splice site. The results from the ultra-deep sequencing approach highlight the impressive fidelity of pre-mRNA splicing and demonstrate that the immediate sequence context around splice sites is the main driving force behind non-canonical splice site pairing.

## Introduction

Splicing is a complex process requiring hundreds of proteins to work in concert with proper regulation [1]. A pre-mRNA transcript from a single gene can be alternatively spliced to generate many mRNA variants. Differential pre-mRNA processing contributes significantly to genetic variability. It is estimated that transcripts from ~86–88% of multi-exon genes undergo alternative splicing [2–4]. Many mRNA isoforms are generated from a single gene as a result of splicing regulation, which may be caused by systemic feedback or tissue-specific expression of splicing regulators [5,6]. Other alternative mRNA isoforms may be the result of erroneous splice site pairing, also referred to as non-canonical splice sites, which may result in the generation of aberrant mRNA isoforms [7]. It has been shown that the most common form of non-canonical splice site activation occurs near the canonical splice site, mainly due to the U1 snRNP binding consensus sequence for 5’ splice sites or duplicate YAG trinucleotides near 3’ splice sites [8,9].

To avoid these errors, there are several safeguards to ensure splicing fidelity. Like transcription and translation, splicing has an active proofreading mechanism, while additionally relying on sequence information to guide the spliceosome through the process. Prp16 [10] and Prp22 [11,12] provide proofreading mechanisms for the first and second sequential transesterification reactions of splicing, and may even remodel the pre-mRNA to activate alternative splice sites [13]. Splicing regulatory element binding sites and the base-pairing of snRNPs to the pre-mRNA substrate lead to the selection of the correct splice sites based on optimal adjacent sequence contexts [14]. Even with these safeguards, splicing fidelity can be compromised when the sequence context for splice sites is suboptimal. All mRNA isoforms are subject to a number of quality control mechanisms, such as nonsense-mediated decay (NMD) [15,16], nonstop decay (NSD) [17], or no-go decay (NGD) [18,19], however, not all aberrant mRNA isoforms are removed through these processes and could be translated.

Owing to the importance of splicing regulation, a large number of mis-splicing or splicing errors can result in different diseases [20]. According to the Human Gene Mutation Database (HGMD release 2014.4), mutations that disrupt normal splicing have been estimated to account for up to a third of all disease-causing mutations [21]. It has been demonstrated that the spliceosome can pair constitutive exons with high fidelity with rates of rare and non-canonical splicing events as low as one in 20,000 [22,23]. Based on these studies, it was suggested that splicing accuracy is limited by Pol II transcription error rates [22,23]. These RT-qPCR based studies are inherently limited to resolution at the exonic level, investigating only single exon skipping events based on genome annotation. Using genome-wide RNA sequencing, similar rates of rare and non-canonical splicing events were observed [24], but it is still unclear whether these aberrant splicing events are the result of transcription errors, poor exon recognition mediated by weak splice sites and splicing regulatory elements, or whether these events are merely stochastic in nature. Additionally, the extent of aberrant mRNA splicing at the nucleotide level remains unknown. Using ultra-deep sequencing we determined the rates of rare and non-canonical splicing events for three splice junctions flanking exon 7 of SMN1 at single nucleotide resolution. We identified previously unannotated splice sites, a potential microexon, potential transcription error-mediated splicing events and the rate at which 5’ splice sites with their inherently susceptible U1snRNP binding site splice at positions 4 nucleotides upstream or downstream of the canonical splice site. Furthermore, we evaluated the effects that mutations in SMN exon 7 have on splicing fidelity.

## Results

### Dataset for ultra-deep analysis of splicing fidelity

**Fig 1:**
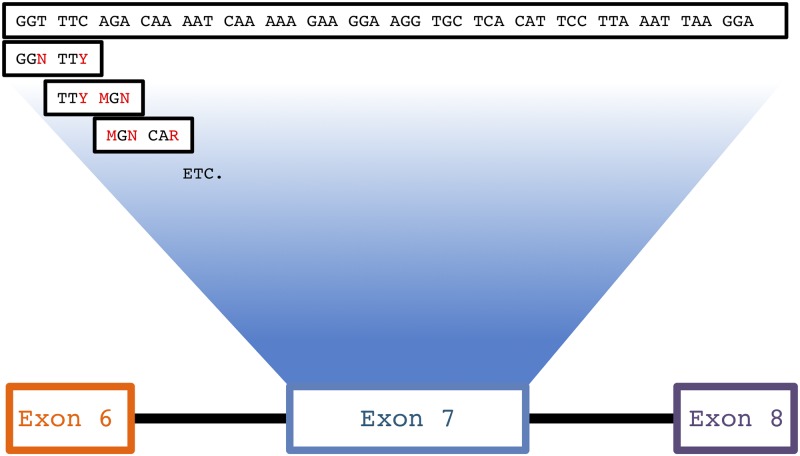
Mutation scheme for SMN1 exon 7 library. The SMN1 mini-gene construct consists of exon 6, exon 7, and exon 8 with shortened introns 6 and 7. All possible silent mutations in exon 7 were created within the context of a sliding hexamer window. For example, the first two codons depicted are GGT TTC. All three mutations were made in GGT resulting in GGN and combined with all silent mutations in TTC (TTT), resulting in eight combinations including the wild-type sequence. A transition 6C→T results in SMN1 exon 7 skipping.

To determine the rates of rare aberrant and non-canonical splicing events we used a recently published dataset [25] of *SMN1* exon 7 inclusion rates based on a synonymous position mutation library in the well-studied SMN1 mini-gene, which spans exons 6–8 [26–28] where exon 7 is included or excluded depending on splicing signals in the pre-mRNA (Fig 1). Neighboring codons in SMN exon 7 were mutated to every possible combination of silent mutations within the context of a sliding hexamer window, a minimal binding site for splicing regulatory proteins [25,29]. The resulting library of plasmids was transfected into HeLa cells and plasmid-specific mRNAs were analyzed by deep sequencing. The data obtained from these library transfections were previously used to determine if synonymous mutations in exon 7 influence splicing. This study also resulted in the several million-fold sequencing of three exon|intron splice site junctions, *SMN1* exon6|exon7, exon7|exon8, and exon6|exon8. This extensive sequencing data allowed for an ultra-deep detection of low abundance local isoforms, including rare stochastic and non-stochastic splicing outcomes described below. Our observations and calculations of splicing fidelity are based on the wild-type *SMN1* mini-gene.

### Total rate of aberrant splicing events

There are several explanations why splicing can occur in a non-canonical way. Here, deviations from the expected canonical exon 7 inclusion splicing pathway (referred to as non-canonical splicing) could be the consequence of imperfections in the generation of the *SMN1* mini-gene mutation library, pre-splicing transcription errors, sequencing errors, or the activation of rarely used splice sites, such as *de novo* splice sites or the selection of microexons. The ultra-deep sequencing of the SMN1 mini-gene highlights several clear-cut examples of non-canonical splice site selection, albeit at a very low rate. Out of a total of 6,469,446 wild-type *SMN1* exon 7 reads 20,505 contained an unexpected alternatively spliced event at either the exon6|exon7 or the exon7|exon8 junction, for a raw aberrant splicing event rate of 3.2E^-03^ or 1 aberrantly spliced event for every 315 splicing events. At first glance this is a higher rate than that of other gene expression steps, transcription and translation, each of which are characterized by error rates as low as 1.0E^-05^ [30–32]. Further examination of the dataset revealed that not every observed deviation from canonical splicing could be counted as a result of aberrant splicing fidelity.

### Control for sequencing errors

**Fig 2:**
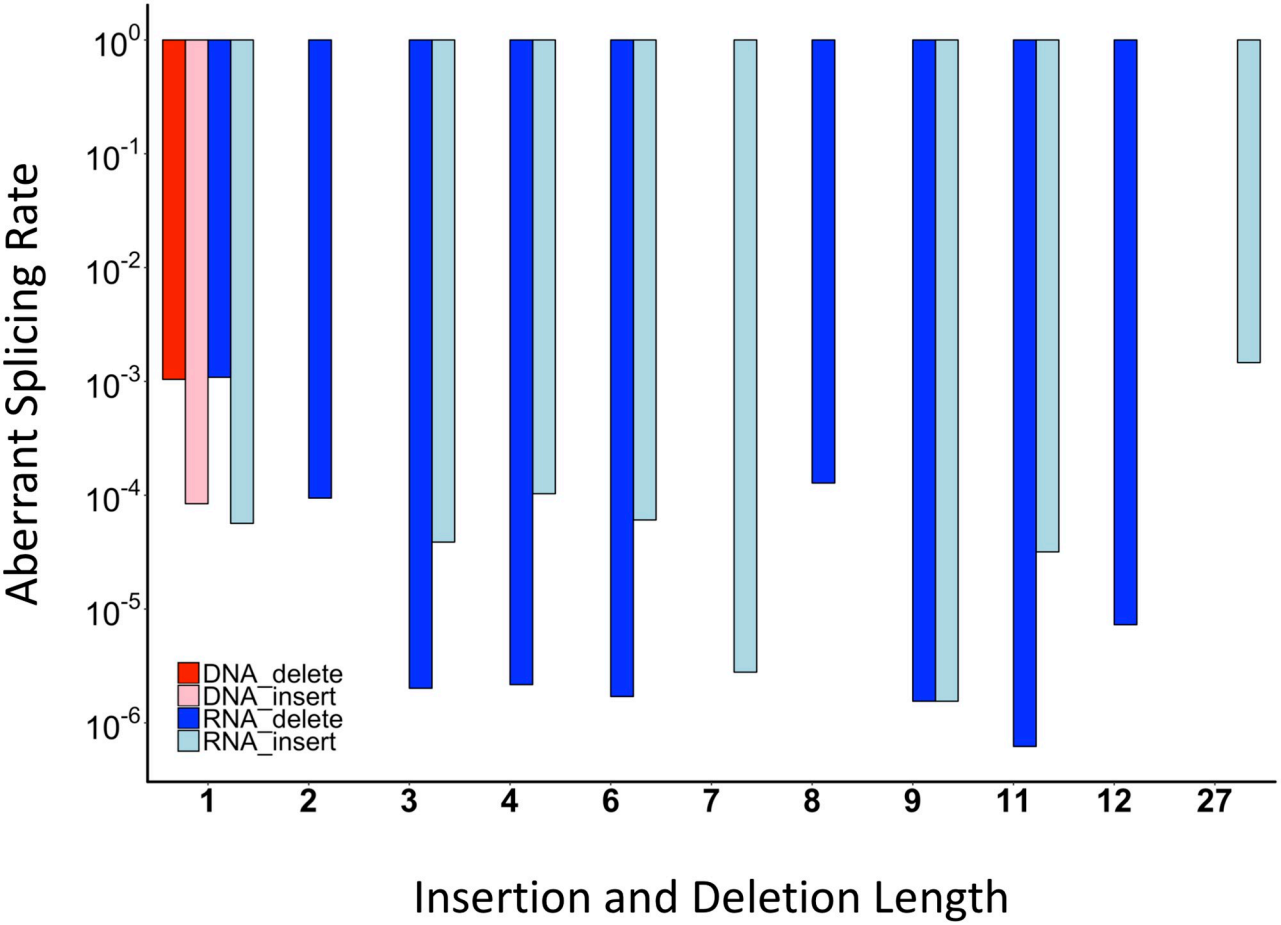
Analysis of aberrant splicing rates in DNA and RNA reads by error length. To detect possible reading frame preservation bias in aberrant splicing events, an analysis of splicing aberrant splicing rates in DNA and RNA by insertion or deletion length in wild-type reads was performed. For example, the insertion of 4 nucleotides GTAA at the 5’SS of exon 7 and the insertion of 4 nucleotides ACAG at the 3’SS of exon 8 are combined as a total aberrant splicing rate for the insertion of 4 nucleotides. There is no consensus error length.

In addition to sequencing the RNA generated from the SMN mini-gene mutation library, the transfected SMN mini-gene DNA constructs were sequenced themselves [25]. The DNA library sequencing served as a control to demonstrate that sequence differences detected in the mRNA reads are due to RNA generation and processing [25]. The most common sequence deviation from the RNA pool was the deletion of a single guanosine from a GGG triplet at the exon6|exon7 junction at a frequency of 1.1E^-03^, accounting for nearly 1/3 of the total identified aberrant splicing events. However, the same deletion occurred within a GGG triplet at a nearly identical frequency at the intron6|exon 7 junction in their DNA counterparts (1.0E^-03^) (Fig 2). Similarly, a single guanosine insertion at this same site, producing a GGGG motif, occurs in 247 DNA reads and 320 RNA reads at rates 8.4E^-05^ and 5.0E^-05^, respectively (Fig 2). These observations strongly suggest that these single guanosine insertions and deletions derive from errors independent of splicing. Importantly, there were no errors in the DNA input reads that resulted in insertions or deletions of multiple consecutive nucleotides. We conclude that any RNA output reads with 2 or more nucleotides consecutively inserted or deleted are attributable to pre-mRNA processing errors or pre-existing sequence variations introduced in the library during its construction.

### Control for plasmid generated errors

**Fig 3:**
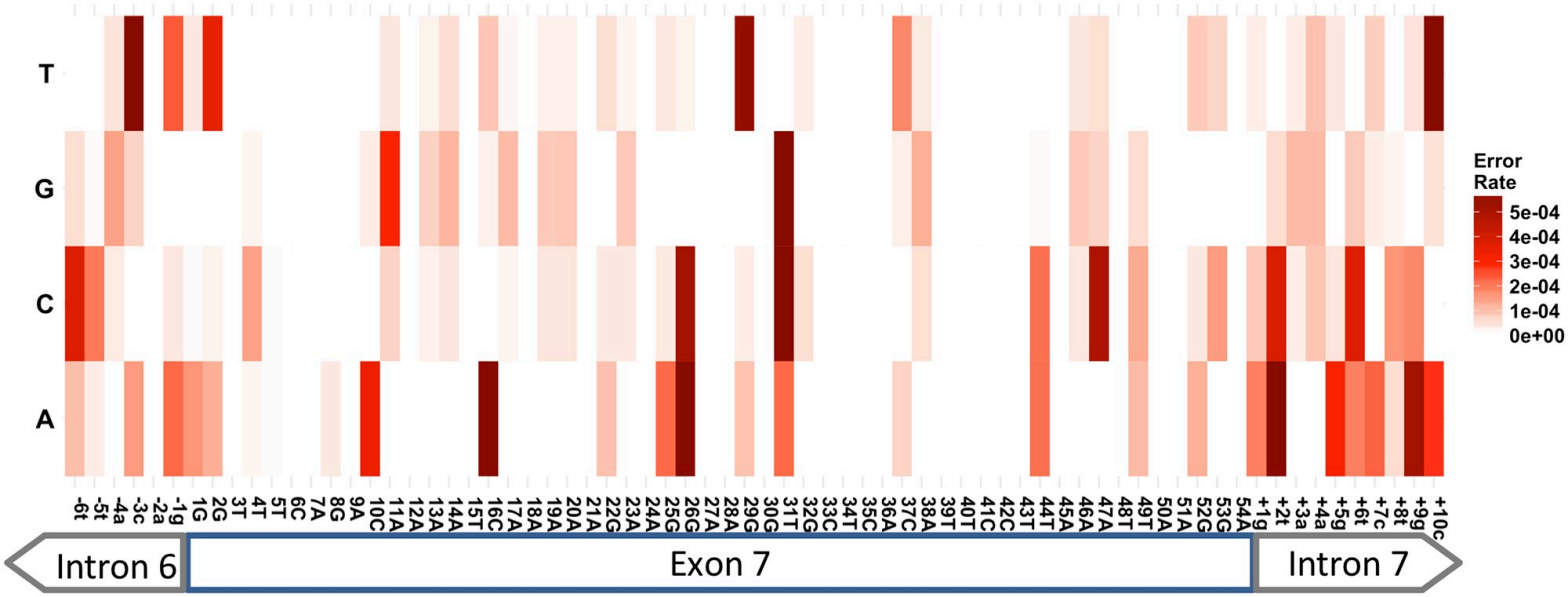
Mutation rate by position in DNA input reads. A heatmap representing positional error rates in DNA input reads that lie within the 15 nucleotide primers on either side of the amplified section that was sequenced (100 nucleotides total). Positions within exon 7 that were purposely mutated in our library construction were omitted and set to zero.

The sequencing of the transfected SMN mini-gene library also serves as a control to demonstrate that errors and aberrantly spliced events identified in the processed RNA reads are due to the generation and processing of the RNA, namely transcription and splicing, and were not already present in the DNA template. Due to the size constraints of the sequencing protocol used (100 nucleotide read length) and the location of the input DNA primers [25], we were only able to estimate the plasmid error rate for the region that was flanked and amplified by the DNA primers used. This region consists of exon7 and the adjacent 6 upstream and 10 downstream nucleotides (Fig 3). While erroneous mutants that arose from errors in the generation of SMN mini-gene DNA construct do exist, they occur at a low rate, averaging 3.0E^-04^ (Fig 3 and Table 1). Although infrequent, these library construction imperfections limit the sensitivity of aberrant splicing detection.

**Table 1:** Average error rate—Input DNA.

	Intron	Exon7	Total
A→C	3.6E^-04^	8.3E^-05^	1.6E^-04^
A→G	7.9E^-04^	1.1E^-04^	3.0E^-04^
A→T	3.9E^-04^	2.8E^-05^	1.3E^-04^
C→A	1.9E^-04^	4.2E^-04^	3.3E^-04^
C→G	4.5E^-05^	2.4E^-05^	3.2E^-05^
C→T	3.6E^-04^	4.2E^-04^	4.0E^-04^
G→A	3.1E^-04^	7.7E^-04^	6.3E^-04^
G→C	8.4E^-05^	9.7E^-05^	9.3E^-05^
G→T	8.5E^-05^	1.4E^-04^	1.2E^-04^
T→A	8.5E^-04^	1.1E^-04^	4.8E^-04^
T→C	3.0E^-04^	1.2E^-03^	7.5E^-04^
T→G	3.9E^-05^	1.9E^-04^	1.1E^-04^

Summary results for each nucleotide substitution across intronic or exonic regions. The intronic region spans the regions 6 nucleotides upstream and the 10 nucleotides downstream of exon 7. The exonic region is based on those nucleotides within exon 7 that were not subjected to synonymous mutation. The calculated number is the error rate of the nucleotide listed first being substituted by the second nucleotide. Total refers to the summation of all substitution errors intronic and exonic.

### Non-canonical 3’-splice site usage

**Fig 4:**
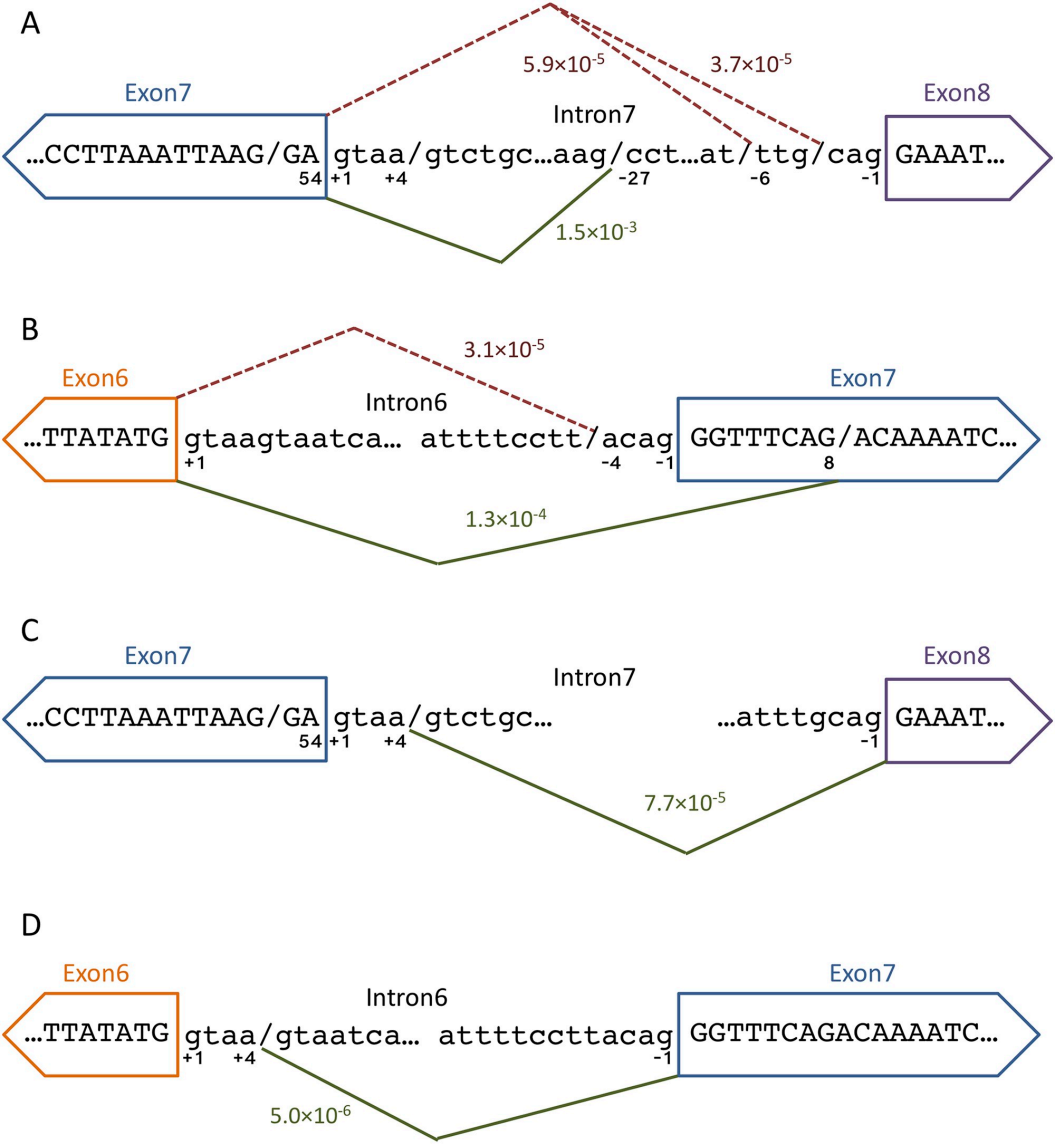
Non-canonical splice site usage. A) Non-canonical 3’SS usage between exon 7 and exon 8. The green line represents the unannotated non-canonical 3’ SS event 27 nucleotides upstream of the canonical intron7|exon8 junction. The red dashed line represents the non-canonical splicing observed that is due to transcription or library generation errors resulting in canonical AG dinucleotide sequences. B) Non-canonical 3’ SS usage between exon 6 and exon 7. The green line represents the unannotated non-canonical 5’ SS event at position 8 in exon 7. The red dashed line represents the non-canonical splicing observed that is due to transcription or library generation errors. C) Non-canonical 5’ SS usage between exon 7 and exon 8. The green line represents the usage of the intrinsic non-canonical 5’ SS. D) Non-canonical 5’ SS usage between exon 6 and exon 7. The green line represents the usage of the intrinsic non-canonical 5’ SS.

An example of abundant non-canonical splicing observed is the selection of an unannotated 3’ splice site 27 nucleotides upstream of the canonical intron7|exon8 3’ splice site (AG/CCTCTGGN_10_…CAG|GA…; where the non-canonical splice site is designated by a “/” and the canonical splice site is represented by a “|”) (Fig 4A). This novel splice site is used at a frequency of 1.5E^-03^ (Table 2) and it is characterized by a canonical AG dinucleotide that defines the 3’ end of nearly every intron in metazoans [33]. However, a poorly defined upstream polypyrimidine tract prevents extensive usage of this non-canonical 3’ splice site (maximum entropy score (MES) = -1.62) [34]. At a splice site usage rate of 1 in 680 transcripts, this non-canonical splicing event is rare enough that it is only readily discovered using ultra-deep sequencing. The upstream location relative to the canonical 3’ splice site polypyrimidine tract suggests that this splice site is acting independent of the canonical 3’ splice site.

**Table 2:** Non-canonical 3’ splice site usage.

Non-canonical Splice Site	Count	Rate	Junction	Type	Location	MES
AG|GGTTTCAG/ACA	825	1.3E^-04^	ex6|ex7	del	down	0.35
AT/TTTCCTTACAG|GGT	202	3.1E^-05^	ex6|ex7	in	up	2.24
AG/CCTCTGGN10…CAG|GAA	9433	1.5E^-03^	ex7|ex8	in	up	-1.62
AT/TTGCAG|GAA	376	5.9E^-05^	ex7|ex8	in	up	6.87
TG/CAG|GAA	238	3.7E^-05^	ex7|ex8	in	up	3.04

The non-canonical splice site is designated by a “/” and the canonical splice site is represented by a “|”. Count refers to the number of wild-type reads that contain the non-canonical splice site, with their associated rate of occurrence. Junction refers to the location of the non-canonical splice site. Type refers to the result of the non-canonical splicing, either an insertion or deletion of sequence from the canonical transcript. Location refers to the position of the non-canonical splice site relative to the canonical splice site. MES is the Maximum entropy score for the cryptic splice site.

At the same intron7|exon8 3’ splice site two additional lower frequency insertions were observed. In 376 cases (aberrant splicing event rate = 5.9E^-05^, Fig 4A) the ligation of exon 7 and exon 8 took place 6 nucleotides upstream of the canonical 3’ splice site (AT/TTGCAG|GAA). The sequence upstream of this non-canonical splice site is an AT dinucleotide, instead of the requisite AG. Thus, the low observed frequency of this non-canonical 3’ splice site selection could be the consequence of selecting a poorly defined AT/TT junction (MES = -1.72), it could have arisen by rare nucleotide mis-incorporation mediated by elongating pol II to change the junction to AG/TT (MES = 6.87), or it could be the consequence of low-level nucleotide variations intrinsic to the mutation library that would also give rise to pre-mRNAs characterized by the improved AG/TT 3’ splice site junction.

To distinguish between these possibilities, we compared the frequency at which alternative nucleotides were detected at invariable nucleotide positions across exon7 of the SMN1 mutant library (Fig 3). On average, the library generation resulted in T to G nucleotide changes at a frequency of 1.1E^-04^ (Fig 3 and Table 1). Using this frequency as a measure for background noise, it is impossible to assign the (AT/TTGCAG|GAA) non-canonical splicing event to any other cause but library generation defects.

At the same intron7|exon8 junction, we also observe 238 similar occurrences (aberrant splicing event rate = 3.7E^-05^, Fig 4A and Table 2) where a possible nucleotide change 4 nucleotides upstream of the canonical 3’ splice site (AT/CAG|GAA → AG/CAG|GAA) drastically changes the favorability of the splice site, (MES = -5.33 → 3.04). These nucleotide changes would create non-canonical 3’ splice sites that approximate those observed in annotated EST databases [8]. Using the same arguments as above, it is most likely that the (AT/CAG|GAA) non-canonical splicing event is observed because of library imperfections.

Analysis of 3’ non-canonical splicing at the exon6|exon7 junction highlights two events that are represented with reasonable frequency. With 825 supporting reads (aberrant splicing event rate = 1.3E^-04^, Fig 4B and Table 2) where the activation of a 3’ splice site 8 nucleotides downstream of the canonical 3’ splice site AG|GGTTTCAG/AC is activated resulting in a truncated exon 7. This non-canonical 3’ splice site has a low MES (0.35), yet its activation does not rely on nucleotide changes at the new spliced junction. Based on these considerations it is likely that non-canonical AG selection intrinsic to the SMN1 wild-type sequence context mediates this non-canonical splicing event.

A lower frequency event is represented by 202 supporting reads (aberrant splicing event rate = 3.1E^-05^, Fig 4B and Table 2) where a non-canonical 3’ splice site (AT/TTTCCTTACAG|GGT) was selected for the intron6|exon7 junction 11 nucleotides upstream of the canonical 3’ splice site. The wild-type sequence upstream of this non-canonical splice site is an AT dinucleotide, instead of the requisite AG, again arguing that the selection of this sequence as a non-canonical splice site is likely a consequence of library imperfections.

### Intrinsic 5’-splice site fidelity—The U1snRNP binding site conundrum

Previous *in silico* sequence analyses have shown that 5’ splice sites are often subject to non-canonical splice site activation 4 nucleotides upstream or downstream from the canonical splice site due to the presence of the U1snRNP binding sequence (AG|**GU**RA**GU**), which commonly includes a GU dinucleotide 4 nucleotides downstream from the canonical splice site [8]. 491 supporting reads reveal the activation of an intrinsic non-canonical exon7 5’ splice site (GA|GTAA/GTCTGC) (aberrant splicing event rate = 7.7E^-05^, Fig 4C and Table 3). While the canonical 5’ splice site is a reasonably strong splice site (MES = 8.57), it should be highly favored compared to this downstream intrinsic 5’ splice site (MES = -7.82).

**Table 3:** Non-canonical 5’ splice site usage.

Non-canonical Splice Site	Count	Rate	Junction	Type	Location	MES
TG|GTAA/GTAATC	32	5.0E^-06^	ex6|ex7	in	downstream	-1.24
GA|GTAA/GTCTGC	491	7.7E^-05^	ex7|ex8	in	downstream	-7.82

The non-canonical splice site is designated by a “/” and the canonical splice site is represented by a “|”. Count refers to the number of wild-type reads that contain the non-canonical splice site, with their associated rate of occurrence. Junction refers to the location of the non-canonical splice site. Type refers to the result of the non-canonical splicing, either an insertion or deletion of sequence from the canonical transcript. Location refers to the position of the non-canonical splice site relative to the canonical splice site. MES is the Maximum entropy score for the non-canonical splice site.

The selection of the non-canonical 5’ splice site at exon6|intron6 (TG|GTAA/GTAATC) was observed only 32 times (aberrant splicing event rate = 5.0E^-06^, Fig 4D and Table 3). This lower rate in non-canonical 5’ splice site activation may be explained by a stronger canonical 5’ splice site (MES = 11.01).

### Microexon discovery in *SMN1*

Another type of rare splicing variants observed are microexons. Many microexons, 3 to 30 nucleotides long, have not been annotated because of their rarity and size [35]. The ultra-deep sequencing analysis identified the presence of a microexon contained within intron 6. The microexon AG/ATCTGGG/GTAATGT is located 210 nucleotides upstream of the intron6|exon7 junction, and it was detected in 18 reads (2.8E^-06^). The microexon is flanked by weak splice sites (3’ splice site, MES = 0.99; 5’ splice site, MES = 4.85) and its usage does not rely on nucleotide changes at the splice junctions. Interestingly, this microexon uses the same 5’ splice site as the recently discovered cryptic exon 7a [36]. Thus, it is possible that the generation of the intron 6 microexon is an alternative splicing pathway in the generation of cryptic exon 7a.

### The influence of exon mutations on splicing fidelity

The library analyzed was created with synonymous mutations at all possible positions within a six-nucleotide window throughout SMN exon 7 [25]. Using the splice efficiency results from this mutant library we tested the hypothesis that positional mutants alter canonical splice-site usage by increasing or decreasing non-canonical splice-site usage. To identify mutations that preferentially influence non-canonical splice-site usage, we focused only on high incidence non-canonical splice events and compared the *Non-canonical Splicing Value* with the published *Inclusion Index Value* [25]. The result of this comparison is referred to as the *Mutant Influence Value*.

### Non-canonical 3’ splice site activation at the exon7|exon8 junction

**Fig 5:**
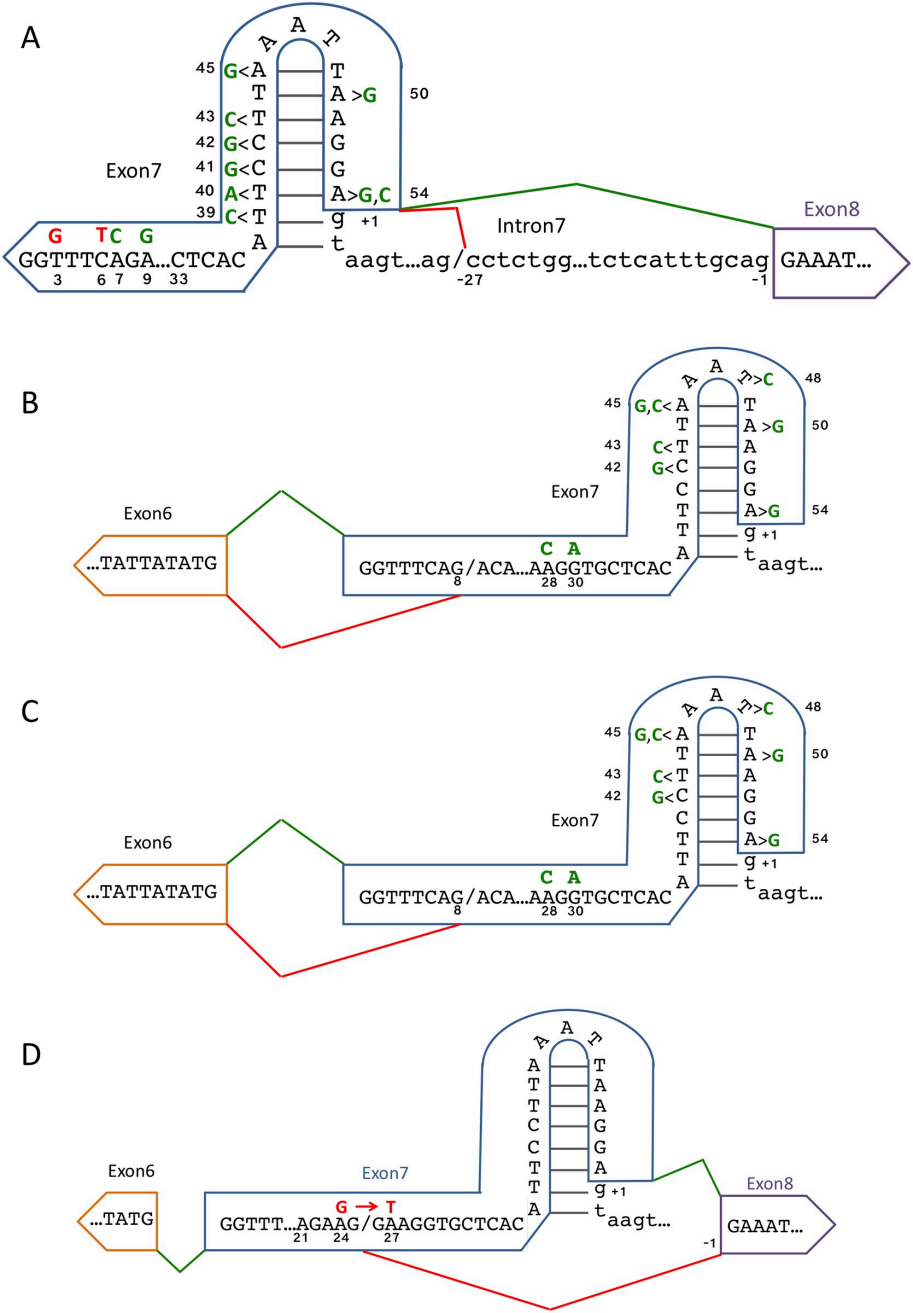
Mutant influence on non-canonical splice site usage. A) Mutant influence on non-canonical 3’SS usage between exon 7 and exon 8. The green line represents canonical SS usage, while the red line represents non-canonical 3’ SS usage 27 nucleotides upstream of the canonical intron7|exon8 junction. Mutations at positions in green influence more canonical SS usage, while mutations at position in red influence more non-canonical SS usage. B) Mutant influence on non-canonical 3’ SS usage between exon 6 and exon 7. The green line represents canonical SS usage, while the red line represents non-canonical 5’ SS usage 4 nucleotides downstream of the canonical intron6|exon7 junction. Mutations at positions in green influence more canonical SS usage. C) Mutant influence on non-canonical 5’ SS usage between exon 7 and exon 8. The green line represents canonical SS usage, while the red line represents non-canonical 5’ SS usage 4 nucleotides downstream of the canonical exon7|intron7 junction. Mutations at positions in green influence more canonical SS usage. D) Mutant at position 27 creates 5’SS. The green line represents canonical SS usage, while the red line represents non-canonical 5’SS usage. Mutations at positions in red influence more non-canonical 5’ SS usage.

A frequently used non-canonical splice site is located upstream of the canonical intron7|exon8 3’ splice site (Fig 4A). Multiple mutations within exon 7 result in altered non-canonical splice site usage, either increasing or decreasing its selection (Table 4). A significant decrease in non-canonical 3’ splice site usage occurs with the mutants 54A→C or 54A→G, which reside within the exon 7 5’ splice site. Combinations of these mutations with 50A→G result in similar effects. Other combinatorial mutations in the region 39 through 45 generally lead to further decreases in non-canonical splice site usage (Table 4 and Fig 5A). Conversely, the combinatorial mutation 3T→G+6C→T results in greater non-canonical 3’ splice site usage. Interestingly, the most influential mutants identified cluster to either the 5’ or the 3’ end of exon 7.

**Table 4:** Mutant influence on splicing fidelity—Non-canonical 3’ splice site AG/CCTCTGGN_10_…CAG|GAA.

Mutation	# Non-canonical Spliced Reads	# Normal Spliced Reads	Inclusion Index Value	Non-canonical Splicing Value	Mutant Influence Value	Mutant Influence Type
Wild-type	9433	6449627	1.0	1.0	0.0	N/A
54A→C	14	35679	1.7	0.2	1.5	SS Strength
54A→G	9	12474	3.3	0.5	2.8	SS Strength
3T→G+6C→T	34	19447	0.1	1.2	-1.1	SRE
7A→C+9A→G	1535	880320	1.6	1.2	0.4	SRE
39T→C+40T→A+41C→G	49	34670	2.2	1.0	1.2	- TSL2
42C→G+43T→C	23	16141	2.3	1.0	1.3	- TSL2
42C→G+43T→C+45A→G	22	10846	2.8	1.4	1.4	- TSL2
42C→G+45A→G	102	49001	2.3	1.4	0.9	- TSL2
50A→G+54A→C	27	41501	1.6	0.4	1.2	SS Strength
50A→G+54A→G	56	75269	3.0	0.5	2.5	SS Strength

Mutant Influence Values are shaded green if the mutation selectively induces canonical splice site usage, white for no splice site preference and the red for non-canonical splice site preference. Significance is set at Benjamini-Hochberg FDR = 0.2. “SS Strength” refers to a change in splice site strength, “SRE” refers to the alteration of a splicing regulatory element, and “- TSL2” refers to the weakening of Terminal Stem Loop 2 within exon 7.

### Non-canonical splicing (GGTTTCAG deletion) at the intron6|exon7 junction

When compared to wild-type non-canonical 3’ splicing at the intron6|exon7 junction AG|GGTTTCAG/AC is significantly reduced by the exon 7 mutations 28A→C, 30G→A, and 45A→G (Table 5 and Fig 5B). As was observed for other non-canonical splicing events, combinations of mutants generally preserve the overall effect single mutants have. By contrast, no mutant significantly increased non-canonical splicing compared to wild-type.

**Table 5:** Mutant influence on splicing fidelity—3’ splice site AG|GGTTTCAG/ACA.

Mutation	# Non-canonical Spliced Reads	# Normal Spliced Reads	Inclusion Index Value	Non-canonical Splicing Value	Mutant Influence Value	Mutant Influence Type
Wild-type	825	6449627	1.0	1.0	0.0	N/A
28A→C	38	519479	1.5	0.5	1.0	SRE
30G→A	82	1378053	1.4	0.4	1.0	SRE
45A→G	6	138179	2.0	0.3	1.7	- TSL2
42C→G+45A→G	5	49001	2.3	0.8	1.5	- TSL2
43T→C+45A→C	3	41164	2.4	0.6	1.8	- TSL2
43T→C+45A→C+48T→C	5	62155	2.3	0.6	1.7	- TSL2
43T→C+45A→G	4	39199	2.5	0.8	1.7	- TSL2
43T→C+45A→G+48T→C	2	39842	2.5	0.4	2.1	- TSL2
50A→G+54A→G	5	75269	2.9	0.5	2.4	SS Strength

Mutant Influence Values are shaded green if the mutation selectively induces canonical splice site usage, white for no splice site preference and the red for non-canonical splice site preference. Significance is set at Benjamini-Hochberg FDR = 0.2. “SS Strength” refers to a change in splice site strength, “SRE” refers to the alteration of a splicing regulatory element, and “- TSL2” refers to the weakening of Terminal Stem Loop 2 within exon 7.

### Non-canonical splicing (GTAA insertion) at the exon7|intron8 junction

Another abundant non-canonical splice site that is affected by mutation is the retention of GTAA by activation of an intrinsic 5’ non-canonical splice site (GA|GTAA/GTCTGC) for exon 7. A significant decrease in non-canonical 5’ splice site usage occurs with the combinatorial mutant 50A→G + 54A→G (Table 6 and Fig 5C).

**Table 6:** Mutant influence on splicing fidelity—5’ splice site GA|GTAA/GTCTG.

Mutation	# Non-canonical Spliced Reads	# Normal Spliced Reads	Inclusion Index Value	Non-canonical Splicing Value	Mutant Influence Value	Mutant Influence Type
Wildtype	491	6449627	1.0	1.0	0.0	N/A
50A→G+54A→G	2	75269	3.0	0.4	2.6	SS Strength
54A→G#	0	12474	3.3	N/A	N/A	SS Strength
50A→G#	7	143757	1.5	0.6	0.9	N/A

Mutant Influence Values are shaded green if the mutation selectively induces canonical splice site usage, white for no splice site preference and the red for non-canonical splice site preference. Significance is set at Benjamini-Hochberg FDR = 0.2. “SS Strength” refers to a change in splice site strength, “SRE” refers to the alteration of a splicing regulatory element, and “- TSL2” refers to the weakening of Terminal Stem Loop 2 within exon 7.

### Mutations create a highly efficient de novo cryptic 5’ splice site

By far the most abundant non-canonical splicing example in the dataset analyzed is the truncation of exon 7 to the first 25 nucleotides. This 5’ non-canonical splicing event (AAG/GAAGGT) is represented by 309,793 reads containing a truncated exon 7 with no other mutations (Table 7 and Fig 5D). However, at a splice site usage rate of 1 in ~21 wild-type transcripts, this non-canonical splicing event is common enough that in the absence of mutation, it should have been readily discovered and annotated without using ultra-deep sequencing. This non-canonical splicing event is most likely the result of one or more mutations downstream of position 25. An analysis of the effects of other mutations on this truncated exon 7 was performed. The difficulty in this analysis is that any mutation that occurs at the 26^th^ through the 54^th^ positions of exon 7 cannot be accurately assessed, as the mutation will be omitted from the read as a result of the exon 7 truncation. The second obstacle in the analysis is that the wild-type reads cannot be used as the comparative baseline. To circumvent these limitations read counts were normalized to the splicing neutral 9A→G [37]. Normalization to splicing neutral 15A→C provided similar results (not shown). The mutation 24A→G occurred in far more truncated exon 7 reads (9,624) than any other mutant (Table 7 and Fig 5D). But the combinatorial mutation 21A→G+24A→G did not occur at an increased rate in truncated exon 7 reads, when compared to splicing neutral mutations 9A→G and 15T→C. These observations suggest that the truncated exon 7 reads containing the mutation 24A→G are actually the result of the 24A→G+27A→T combinatorial mutant (and probably to a lesser extent 24A→G+27A→C), as these mutations exhibit the same exon 7 inclusion behavior as the single mutations 27A→T and 27A→C in a previous study [25].

**Table 7:** Mutation-derived non-canonical 5’ splice site at position 27 results in truncated exon 7.

Mutation	# Non-canonical Spliced Reads	# Normal Spliced Reads	Inclusion Index Value	Non-canonical Splicing Value	Mutant Influence Value	Mutant Influence Type
Wild-type	309793	6449627	1.1	218.5	-217.4	5’SS
21A→G+24A→G#	9	145745	1.2	0.3	0.9	N/A
9A→G	230	1046328	1.0	1.0	0.00	N/A
24A→G	9624	289440	0.8	151.3	-150.5	5’SS

Mutations at position 27 create a highly efficient non-canonical 5’ splice site. Calculations are based on splicing neutral 9A→G instead of wild-type. Mutant Influence Values are shaded green if the mutation selectively induces canonical splice site usage, white for no splice site preference and the red for non-canonical splice site preference. Significance is set at Benjamini-Hochberg FDR = 0.2. 5’SS refers to the creation of highly efficient 5’SS. # refers to “not statistically significant.”

### Non-canonical splicing during skipping of exon 7

**Fig 6:**
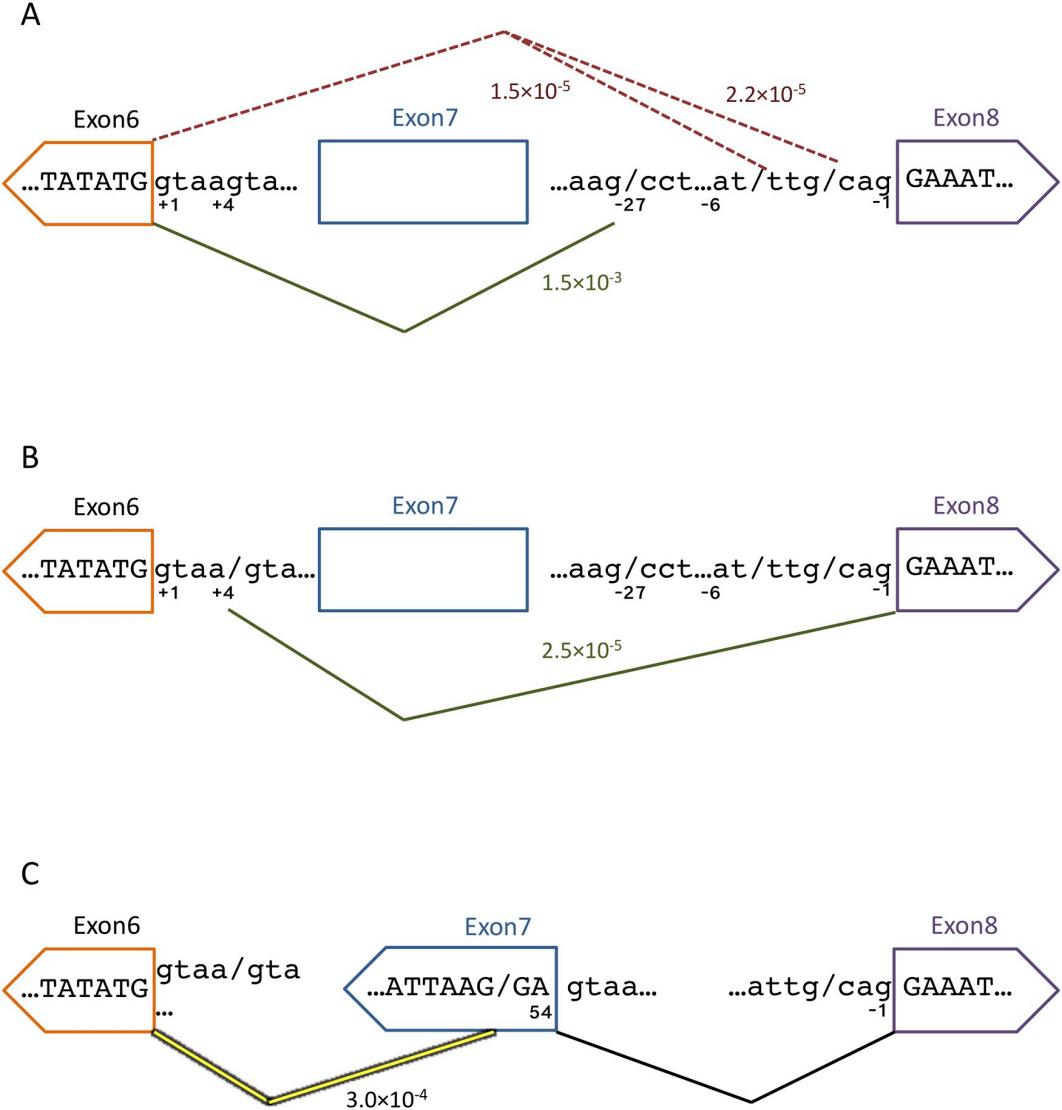
Non-canonical splice site usage with excluded exon 7. A) Non-canonical 3’SS usage between exon 6 and exon 8. The green line represents the unannotated non-canonical 3’ SS event 27 nucleotides upstream of the canonical intron7|exon8 junction. The red dashed line represents the non-canonical splicing observed that is due to transcription or library generation errors resulting in canonical AG dinucleotide sequences. B) Non-canonical 5’ SS usage between exon 6 and exon 8. The green line represents the usage of the intrinsic non-canonical 5’ SS. C) Recursive splicing resulting in GA dinucleotide insertion between exon 6 and exon 8.

The mini-gene used in our analysis also generates mRNA transcripts with skipped exon 7. We recovered ~4 million reads that include exon 6 and exon 8 but skip exon 7 in the dataset analyzed. While we are unable to directly validate the junctions observed in these skipped exon 7 reads, they can be compared to the exon 7 inclusion reads. In general, exon 7 exclusion events contain the same rare and non-canonical splicing events at similar rates. The non-canonical 3’ splice site upstream of exon 8 (AG/CCTCTGGN_10_…CAG|GA…) is observed at a frequency identical to the usage rate seen for exon 7 inclusion events (1.5E^-03^ vs 1.5E^-03^) (Tables 2 and 8, Fig 6A). Other non-canonical splice site selection events at the exon 6|intron 6 or the intron7|exon8 junctions are also observed at similar frequencies (Tables 2, 3 and 8, Fig 6A and 6B). A surprising result in the analysis of exon 7 exclusion transcripts was the discovery of a GA dinucleotide frequently inserted between exon 6 and exon 8 (1257 occurrences, aberrant splicing event rate = 3.0E^-04^, Table 5). As this microexon event occurs at a higher rate than our established library construction error rate, it is highly likely that this event is the result of non-canonical splice site usage (Fig 6C).

**Table 8:** Skipped exon 7 cryptic splicing.

Non-canonical Splice Site	Count	Rate	Junction	Type	Location
AG/CCTCTGGN10…CAG|GAA	6372	1.5E^-03^	ex6|ex8	in	up
AG/GA|GTAAGT	1257	3.0E^-04^	ex6|ex8	in	recursive
TG|GTAA/GTAATC	107	2.5E^-05^	ex6|ex8	in	down
TG/CAG|GAA	92	2.2E^-05^	ex6|ex8	in	up
AT/TTGCAG|GAA	63	1.5E^-05^	ex6|ex8	in	up
AG/ATCTGGG/GTAATGT	3	7.0E^-07^	ex6|ex8	in	microexon

The non-canonical splice site is designated by a “/” and the canonical splice site is represented by a “|”. Count refers to the number of wild-type reads that contain the non-canonical splice site, with their associated rate of occurrence. Junction refers to the location of the non-canonical splice site. Type refers to the result of the non-canonical splicing error, either an insertion or deletion of sequence from the canonical transcript. Location refers to the position of the non-canonical splice site relative to the canonical splice site.

## Discussion

### Non-canonical splicing detection is limited by sequence context and sequencing accuracy

The spliceosome has evolved several mechanisms to ensure that the intron/exon junction is selected efficiently and accurately. Base pairing between U1 snRNA and the 5’ splice sites ensures proper selection of the exon/intron junction. In this process LUC7, a component of yeast U1 snRNP, stabilizes its interaction with the 5’ splice site [38]. During spliceosomal assembly active proofreading mechanisms through the activities of Prp16 [10] and Prp22 [11,12] ensure that the first transesterification reaction of splicing is carried out faithfully. At the 3’ splice site interactions of U2AF with the polypyrimidine tract and U2 snRNP with the branch site are essential for the recognition of the intron/exon junction [39]. While the small subunit of U2AF can initially interact with the AG dinucleotide at the 3’ splice site, hSlu7 and SPF45 select the AG dinucleotide prior to the second step of splicing [40,41] while Prp8 conformational changes align the upstream exon with the AG dinucleotide in the active site [42]. Together, these selection mechanisms ensure the two-step excision of introns and ligation of exons is carried out accurately.

The most common spliced sequence deviation identified in the dataset analyzed was the deletion of a single guanosine from a GGG triplet at the exon6|exon7 junction. However, this G insertion likely reflects sequencing errors because G insertions observed in control DNA reads occurred at an identical frequency. The GGG sequence context is more prone to Illumina HiSeq sequencing errors when compared to genome-wide observations [43], thus explaining the abundance of errors observed. These considerations support the idea that the numerous guanosine insertion/deletion events found at the intron6|exon7 junction are mainly attributable to sequencing errors inherently associated with GGG motifs.

Within the context of the mini-gene used the most abundant non-canonical splice sites used are located close to 3’ splice sites. This may be due to the two-step process of 3’ splice site selection described above. It is possible that these consecutive selection steps at the 3’ splice site are more prone to spurious splice site selection when compared to the base pairing guided selection of the 5’ splice site.

Despite the sequencing depth across the three junctions evaluated, we did not find evidence for multiple non-canonical splice events within a single transcript. This observation suggests that upstream non-canonical splicing does not increase the likelihood of downstream non-canonical splicing, at least as far as our mini-gene approach can decipher. Considering the locations of non-canonical splicing, the results of the ultra-deep sequencing analysis demonstrate that non-canonical splicing usage is constrained by the sequence surrounding putative splice sites and that non-canonical splicing is more likely to occur at 3’ splice sites.

### The reading frame is not preserved by non-canonical 5’ splice site selection

It was argued that the SMN1 mini-gene used for generating the ultra-deep sequencing dataset is not subject to mRNA surveillance methods such as NMD, as it lacks the features of a full-length coding transcript [16,25]. This independence from of frameshift considerations allowed the generation and detection of all possible non-canonical splicing events. As expected from the independence of NMD, we do not detect a direct reading frame preference for the aberrant splicing events observed. Thus, the splicing events observed, non-canonical or canonical, are all a result of a contextual sequence dependent process not beholden to frame preservation.

Previous work postulated that the activation of downstream non-canonical 5’ splice sites at the +4 position are subjected to less selective pressures because they are not part of the coding region. The results from our analysis are in agreement with this notion. Only non-canonical 5’ splice sites located within the canonical intronic region were selected at a statistically significant level. Within the native context of *SMN1* exon 7, an intronic +4 non-canonical 5’splice site will be positioned downstream of the designated stop codon, which is located in exon 7. Thus, the lengthening of exon 7 by 4 nucleotides through non-canonical splicing would not disrupt the coding region of the gene, nor would the non-canonical transcript isoform be subject to NMD. These considerations suggest that evolutionary pressures to prevent non-canonical +4 splicing at the exon 7 5’ splice site are less stringent. Indeed, our findings support this hypothesis because the number of non-canonical 5’ spliced events at exon 6, which is entirely coding, is 10-fold less frequent than those detected for exon 7.

### Skipped exon 7

While the preferential usage of non-canonical splicing is the result of several different mutant influences, the fact that they occur at similar frequencies in exon 7 inclusion and exclusion types suggests that splice site selection occurs at each splice site independently. The most surprising result in the analysis of exon 7 exclusion transcripts was the discovery of a GA dinucleotide frequently inserted between exon 6 and exon 8 (Table 8 and Fig 6C). While there are 66 instances of GA dinucleotides in the intervening sequence between exon 6 and exon 8, only four are flanked by a GT dinucleotide (GA/GT) synonymous with 5’ splice sites. Additionally, an AG dinucleotide, generally associated with 3’ splice sites (AG/GA), positioned next to the GA dinucleotide occurs five times. Only one of these prospective GA dinucleotides is flanked by both an AG at the potential 3’ splice site and a GT at the presumed 5’ splice site (AG/GA/GT). This single prospective GA dinucleotide uses the canonical 5’ splice site for exon 7, while the hypothetical 3’ splice site generates a respectable splice site strength score (MES = 4.28). Thus, while this GA dinucleotide insertion event cannot be unambiguously mapped, the hypothetical splice site evidence flanking the singular AG/GA/GT site strongly suggests that this microexon is a result of the non-canonical splicing of exon 7. This unexpected isoform may be generated through a form of recursive splicing, where all but the last two nucleotides of exon 7 are lost to a non-canonical 3’ splice site (AAG/GA|GTAAGT) contained at position 52. It is possible that this non-canonical 3’ splice site is selected and once intron 6 and the first 52 nucleotides of exon 7 are excised, the last two nucleotides of exon 7 are ligated to exon 6 and redefined as part of a 5’ splice site prior to removal of intron 7.

### Splicing fidelity is a sequence driven process influenced by splicing efficiency

Three abundant non-canonical splice sites altered usage rates when tested in mutant contexts. Mutational effects on non-canonical 3’ splicing at the exon7|exon8 junction (Table 4 and Fig 5A) can be explained by three separate factors that are expected to alter the splicing efficiency. The first factor is the manipulation of splicing regulatory elements within exon 7. The exon 7 mutant 3T→G+6C→T was implicated as a mutation that affects non-canonical splice site usage and greatly reduces exon 7 inclusion [25]. It is well known that the mutation 6C→T in *SMN1* results in decreased inclusion of exon 7 [26,44] by modulating the binding affinity of splicing regulators [45,46]. Combinatorial mutations at position 3T→A or G synergize with 6T to further decrease exon 7 inclusion levels [25,47]. What is interesting is that this mutation, which is located near the 5’ end of exon 7, has such a marked effect on downstream non-canonical 3’ splice site usage. These observations suggest that elevated non-canonical splicing during intron 7 removal is triggered by inefficient exon 7 removal, a demonstrated consequence of the 3T→G+6C→T mutation.

The second factor affecting non-canonical splicing at the exon7|exon8 junction is RNA secondary structure. All significant combinatorial mutations located between 39 and 45 lie within the reported exon 7 inhibitory terminal stem-loop 2 (TSL2) [28]. All mutations are predicted to simply disrupt the stability of this inhibitory RNA hairpin [28,48], thereby promoting the canonical splicing pathway (Fig 5A).

The third factor involved in the mutational influence on non-canonical 3’ splicing at the exon7|exon8 junction is the direct altering of splice site strength. The reduced selection of non-canonical splicing for mutations 54A→C and 54A→G (Table 4 and Fig 5A) can be explained by an increase in exon 7 5’ splice site strength (wild-type MES = 8.57 increases to 54A→C (MES = 9.39) and 54A→G (MES = 9.65)). The strengthening of the 5’ splice site on exon 7 provides increased exon definition, thus favoring the canonical pathway.

Non-canonical 3’ splice site selection at the intron6|exon7 junction can be explained by the same three factors, modulation of SREs, changes in RNA secondary structure and changes in splice site strength (Table 5 and Fig 5B). Mutations at nucleotide positions 28–30 reside within a conserved tract of exon 7 [28] that is directly adjacent to a Tra2-β1 binding site [49], a splicing enhancer [50,51]. Disruption of the TSL2 RNA secondary structure by combinatorial mutations between positions 42 and 50 generally decreases non-canonical splicing at the exon6|exon7 junction, presumably because these mutations increase the efficiency of canonical splicing. Similarly, splice site mutations (positions 50–54) increase canonical splicing and decrease non-canonical splicing.

The only mutation that significantly influences non-canonical 5’ splice site usage in exon 7 is 50A→G+54A→G (Table 6 and Fig 5C). Its appearance as a mutation significantly altering non-canonical 5’ splice site usage indicates the importance of splice site strength on splicing efficiency and its effect on splicing fidelity. As was argued above, increasing the strength of the canonical 5’ splice site results in greater inclusion of exon 7, and decreased usage of the non-canonical splice site.

In summary, changes in the relative usage of non-canonical splice sites can be explained by its inverse relationship with the efficiency of canonical splicing. Mutations that increase canonical splice site selection reduce non-canonical splice site activation. By contrast, mutations that reduce canonical splice site recognition increase non-canonical splice site selection by modulation of SREs, changes in RNA secondary structure and changes in splice site strength.

### Mutation at position 27 creates a highly efficient non-canonical 5’ splice site

While the influence of the previously mentioned mutations on non-canonical splicing are straightforward, the most abundant non-canonical splicing event is more difficult to decipher. Although we detected 309,793 reads as having a non-canonical splice site at position 25 resulting in a truncated exon 7, this non-canonical splice event was not a previously annotated splice site. We propose that this non-canonical splicing event is the result of the creation of a *de novo* non-canonical 5’ splice site by the mutation 27A→T (Table 7 and Fig 5D), which creates a strong 5’ splice site AAG/G**T**AGGT (MES = 10.29). These results demonstrate how splicing efficiencies can be radically changed under the influence of a single mutation.

In summary, the ultra-deep sequencing analysis provides evidence for the notion that the balance between non-canonical and canonical splicing is determined by their relative usage efficiency, a concept that extends beyond the SMN1 model used here. Several factors, including splice site strength, binding sites for splicing regulators and RNA secondary structure play an active role in establishing a preference between competing splicing kinetics. Single mutations within exons can significantly disturb this balance, leading to non-canonical splice site activation within the exon affected or its flanking introns.

## Materials and methods

### Cell culture, sequencing library preparation

The creation and sequencing of the mutant library was executed by our lab [25], in brief, HeLa cells were used for creation and transfection of the SMN1 Exon 7 mutant library. These were maintained in monolayer at 37°C in Dulbecco’s high glucose modified Eagle’s medium (Invitrogen) supplemented with 10% fetal bovine serum, 4mM L-Glutamate, and 1mM Na-Pyruvate. Cell confluence was maintained at ~80% or less before splitting cells. Cells were transfected according to manufacturer’s specifications for Lipofectamine 2000 (Invitrogen) for plate sizes of 10cm, 15cm, and 6-well plates with 3cm wells.

### Bioinformatic analysis of splicing fidelity for *SMN1* mini-gene

We obtained 54,780,073 single-end reads of 100 nucleotides from the sequencing run. These reads were aligned to a custom index consisting of genomic SMN1 exons 6 to 8, spliced mRNA sequence consisting of exons 6, 7, and 8, and all exon 7 mutants that were introduced into the library. The reads were classified as either input reads, which would be the unprocessed DNA based reads, and output reads which would comprise all reads that were sequenced from processed mRNAs, which would include undergoing transcription and splicing. We used custom Python scripts to identify reads with wild-type exon 7 and all mutant exon 7 types. Regular expression search functions were employed to search for multiple anchor sequences associated with exon 6, exon 7, exon 7 mutants, and exon 8. In order to determine the existence of aberrant splicing events we checked each read that contained these anchor sequences for either unexpected additional sequence inserted between exon anchor sequences or sequence deleted from the expected anchor sequences that result in partial anchor sequences. Aberrant splicing event rates were calculated as percentages where the total number of normal reads divided by the total number of reads that contained each distinct event. Reads where anchor sequences for exon 6 and exon 8 were found, but no anchor sequences from exon 7 were considered to be non-canonically spliced reads where exon 7 was excised. Reads that did not contain anchor sequences or with multiple quality score based errors resulting in ambiguous nucleotides were discarded.

### Mutation position effects on splicing fidelity

There are several examples of mutations that increase non-canonical splicing. However, many of these mutations also increase wild-type exon 7 inclusion levels, confounding the number of increased non-canonical splice site usage reads with an increased total number of exon 7 inclusion reads. For instance, mutations at positions 42C→T+43T→C+45A→G has a *Non-canonical Splicing Value* of 2.0 for splicing of AG/CCTCTGGN_10_…CAG|GA at the intron7|exon8 junction. However, this same set of mutations is also responsible for a 2.1-fold increase in exon 7 inclusion according to its *Inclusion Index Value* [25]. Therefore, the increased number of reads containing non-canonical splicing compared to wild-type is inherently tied to the increase of exon 7 inclusion by this same set of mutations.

To calculate the effect of mutations on splicing fidelity, taking into account the rarity of events, we utilized odds ratios (OR) to determine those mutations that significantly change the ratios of aberrant splicing events compared to the aberrant splicing event rates observed in the wild-type SMN exon 7. We calculated the OR for each SMN1 exon 7 mutant type by taking the rate of each distinct non-canonical splicing event and divided it by the rate that the corresponding non-canonical splicing event occurs in wild-type SMN1 exon 7. This we refer to as the *Non-canonical Splicing Value*. To normalize the influence of exon 7 inclusion rates on the *Non-canonical Splicing Value*, we took the difference between the published *Inclusion Index Value* [25] creating the *Mutant Influence Value*. We then took the absolute value of the *Mutational Influence Value* and calculated the standard error. A z-score statistic was calculated and used to determine the p-value for the difference between the *Non-canonical Splicing Value* and the *Inclusion Index Value*. To account for multiples testing problems, the Benjamini-Hochberg procedure was used at a level of 0.2, to control the false discovery rate. Additionally, a minimum of 10 non-canonically spliced reads (based on the wild-type non-canonical splicing rate) threshold was imposed to avoid outsized conclusions based on small sample size.
